# Iron Biofortification of Staple Crops: Lessons and Challenges in Plant Genetics

**DOI:** 10.1093/pcp/pcz079

**Published:** 2019-05-06

**Authors:** James M Connorton, Janneke Balk

**Affiliations:** 1 Department of Biological Chemistry, John Innes Centre, Norwich, UK; 2 School of Biological Sciences, University of East Anglia, Norwich, UK

**Keywords:** Crop, Mineral, Nutrition, Phytate

## Abstract

Plants are the ultimate source of iron in our diet, either directly as staple crops and vegetables or indirectly via animal fodder. Increasing the iron concentration of edible parts of plants, known as biofortification, is seen as a sustainable approach to alleviate iron deficiency which is a major global health issue. Advances in sequencing and gene technology are accelerating both forward and reverse genetic approaches. In this review, we summarize recent progress in iron biofortification using conventional plant breeding or transgenics. Interestingly, some of the gene targets already used for transgenic approaches are also identified as genetic factors for high iron in genome-wide association studies. Several quantitative trait loci and transgenes increase both iron and zinc, due to overlap in transporters and chelators for these two mineral micronutrients. Research efforts are predominantly aimed at increasing the total concentration of iron but enhancing its bioavailability is also addressed. In particular, increased biosynthesis of the metal chelator nicotianamine increases iron and zinc levels and improves bioavailability. The achievements to date are very promising in being able to provide sufficient iron in diets with less reliance on meat to feed a growing world population.

## Introduction

Biofortification of staple crops is widely considered a sustainable and long-term approach to ameliorate nutrient deficiencies. In particular iron, zinc, selenium and vitamin A are the focus of biofortification programs around the globe, with the aim of complementing and in some cases replacing chemical fortification or food supplementation. Iron deficiency is the most prevalent and widespread nutrient deficiency (WHO 2017). It is the main cause of anemia (∼50% of cases), and associated with poor pregnancy outcome, impaired cognitive development, lower immunity and reduced work productivity resulting from tiredness (WHO 2017). While other factors can indirectly lead to iron deficiency, the main cause is low iron intake from diets consisting predominantly of starch-rich, but nutrient poor, staple crops such as white rice, corn meal, wheat flour, potatoes or cassava. While wholegrain cereals have a similar iron concentration to meat products (Table�1), the iron is primarily found in the aleurone and embryo (Fig.�1) and these tissues are removed by postharvest processing (polishing rice and milling of white flours). Moreover, the bioavailability of mineral nutrients in plant foods is significantly lower than in meat because of the presence of antinutrients such as polyphenols and phytic acid in plants (Hurrell and Egli 2010).

**Fig. 1:**
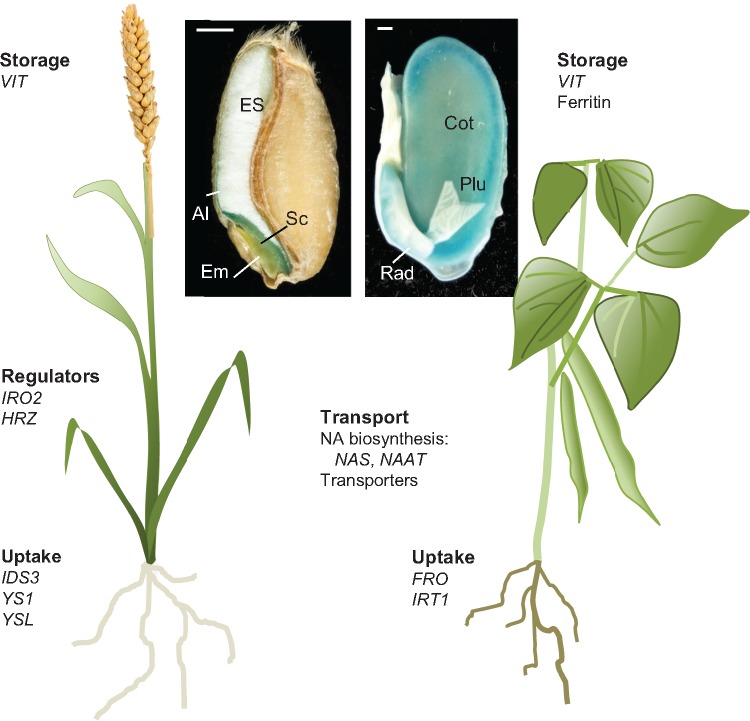
Physiological processes of iron homeostasis and genes used for iron biofortification. Cross sections of a wheat grain (*Triticum aestivum*) and common bean seed (*P. vulgaris*) are shown after staining for iron with Perls’ staining (blue) to show the fundamentally different distribution of iron in these two seed types. Al, aleurone; Em, Embryo; ES, endosperm; Sc, Scutellum; Cot, cotyledon; Plu, plumule with first true leaves; Rad, radical. Scale bar is 1 mm.

**Table 1:** Iron concentrations in plant foods

Plant food	Typical iron concentration (�g/g)^a^	Natural variation in iron concentration (mg/g DW) (*n* lines)	Biofortification target set by HarvestPlus (�g/g DW)^b^	Fold increase
Rice, brown	15	1–26.8 (274)^c^		
Rice, polished	2	4–30 (285)^d^	15	7.5�
Wheat, wholemeal	30	26.3–68.8 (600)^c^	59	2�
Wheat flour, white	7	5.5–15.7 (43)^e^		
Maize, whole	30	11.3–60 (30)^c^	60	2�
Common bean	50	35–93 (1072)^d^	107	2.1�
Peas, dried	50	23–105 (481)^d^		
Pearl millet	47	19.7–86.4 (225)^b^	88	1.9�
Cassava root	5	6–230 (600)^d^	45	9�
Sweet potato	6	3.2–16.0 (12)^f^	85	14.2�
Irish potato	3	30–156 (74)^g^		
Cabbage, broccoli	17			
Tomatoes	5			
Beef steak	35			

^a^All values are per gram of the purchased products, which is ‘wet weight’ for cassava, potatoes and vegetables. The iron concentrations in this column can be lower than the values for natural variation in the next column, which are always reported per gram dry weight (FSA 2002).

^b^ Bouis et al. (2011).

^c^ Goudia and Hash (2015).

^d^ White and Broadley (2009).

^e^ Tang et al. (2008).

^f^ Laurie et al. (2012).

^g^ de Haan et al. (2012).

The daily requirement for iron (median, absolute values) is 0.71 mg/day for children (7–10 years old), 1.05 mg/day for adult men and 1.46 mg/day for adult women (FAO/WHO 2004). Based on these values, as well as the per capita consumption of staple crops, mineral loss during food preparation and estimated bioavailability, the desired iron concentration for specific staple crops has been calculated (Bouis et�al. 2011; Table�1). For crops such as pearl millet and common bean, values close to these targets have been achieved by breeding programs, and there is good evidence from nutrition intervention studies that iron-biofortified crops can indeed increase iron status of target groups (reviewed in Lockyer et�al. 2018). However, there is too little genetic variation in iron concentration in the endosperm of cereal grains (especially rice, wheat and corn), therefore transgenic approaches may be the only possible way of obtaining varieties with increased iron.

Research into mineral biofortification has accelerated with the availability of genome sequences, enabling a full experimental cycle of forward and reverse genetics approaches (Fig.�2). Advances in genome-sequencing technology have made it much less time-consuming and cheaper to zoom in on genes underlying quantitative trait loci (QTL), or to identify these genes directly using genome-wide association studies (GWAS). At the same time, >10 years of transgenics studies, using iron homeostasis genes from model organisms and altering their expression in staple crops, have provided invaluable insight into strategies for biofortification.

**Fig. 2:**
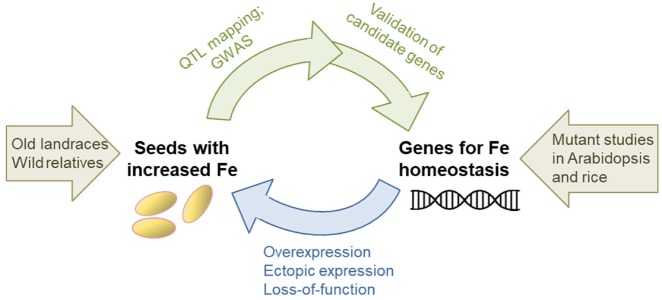
Different research strands enabling iron biofortification of crops. With the revolution in genome sequencing, forward genetics approaches such as QTL mapping and GWAS facilitate the discovery of genes involved in iron homeostasis. Whether polymorphisms in ‘candidate genes’ are the cause of higher iron levels can be verified by using TILLING mutants or gene editing. At the same time, genetic markers in high-iron loci can be used for breeding purposes. In reverse genetics approaches, the expression of known iron homeostasis genes is manipulated to increase the iron concentration of seeds.

## Increasing Iron by Conventional Breeding

Food crops have been selectively bred for centuries to have desirable traits. The amount of available genetic diversity for a particular trait, which can be exploited by breeding, varies depending on the crop (see Table�1 for iron). The genetics underlying some traits, such as pro-vitamin A accumulation, are well understood and have led to the breeding of crops such as maize rich in pro-vitamin A (Gebremeskel et�al. 2018). For many other traits, including high iron, progress has been slower. Iron homeostasis in plants is tightly regulated, and biofortification often requires circumventing these regulatory mechanisms to allow iron accumulation in a target tissue (Connorton et�al. 2017a). While transgenic strategies (see below) target specific genes known to play a role in iron homeostasis, traditional breeding relies on inheritance of the high-iron phenotype, together with a particular genetic marker (Rommens 2007).

There is often less genetic diversity in modern cultivars of most crops compared with older landraces and wild ancestors (Esquinas-Alc�zar 2005). For example in wheat, the iron levels of modern cultivars are relatively low and the downward trend in iron concentration is continuing as yield increases (Fan et�al. 2008). There is also evidence that environmental factors, such as increasing atmospheric CO_2_ are likely to lead to a further decline in iron in wheat (Myers et�al. 2014). While traditional varieties of rice contain higher concentrations of iron than modern varieties, this may be associated with a yield penalty (Anandan et�al. 2011). Nevertheless, there is currently much interest in introducing desirable traits through the introgression of alleles from wild ancestors to modern crops (Palmgren et�al. 2015). This has been successful in improving the concentration of iron in grains, e.g. the introgression of chromosomal regions from wild ancestor *Aegilops* species into modern wheat has led to more than doubling of grain iron (Tiwari et�al. 2010, Neelam et�al. 2011).

GWAS and QTL mapping have been extremely useful in identifying chromosomal regions, and even specific alleles, associated with high iron in crops. GWAS is able to assess the effect of many different single nucleotide polymorphisms (SNPs) in an unrelated population (Mitchell-Olds 2010). It is useful in determining the effect of polymorphisms in a specific gene, and can help in identifying individual genes playing a role in increasing iron levels. QTL mapping, however, takes advantage of large mapping populations available in many crops, including rice and wheat, and is a powerful tool for studying polygenic, quantitative traits (Collard et�al. 2005). Due to the relative scarcity of genetic markers in the genome, QTL often correspond to large chromosomal regions, with sometimes over a hundred genes. Refining the region and identifying particular genes involved can be a challenging next step, and a combination of QTL mapping and GWAS aids this process. Furthermore, as the effect of a particular QTL can be modulated by environmental factors and vary dramatically between studies (Garcia-Oliveira et�al. 2018), meta-QTL analysis is a useful tool for combining and integrating information from different studies and can shed light on the genetic architecture behind a particular trait (Wu and Hu 2012). In common bean (*Phaseolus vulgaris*), a meta-QTL analysis revealed two QTL associated with high iron, and a further eight QTL associated with combined high iron and zinc, across seven studies in South and Middle America (Izquierdo et�al. 2018). A total of 12 candidate genes were identified in these QTL, belonging to families including metal transporters (*NRAMP*, *MATE* and *ZIP*), ferric chelate reductases and a bZIP transcription factor (Izquierdo et�al. 2018).

Through GWAS of a 144-strong Multi-parent Advanced Generation Inter-Cross (MAGIC) Plus population, loci associated with grain iron concentration have been identified in rice, including known iron homeostasis genes such as nicotianamine (NA) synthase *OsNAS3* and vacuolar iron transport *OsVIT1* (Descalsota et�al. 2018). In wheat, a recent GWAS study identified 137 SNPs associated with a significant difference in grain iron concentrations, which ranged from 24 to 52 mg/kg (Alomari et�al. 2018). The identity and specific functions of many of the SNP-containing genes remains to be determined, however, some are located in a NAC family transcription factor. These play a key role in mineral remobilization in wheat and are very important in determining the nutrient content of grains (Uauy et�al. 2006).

QTL mapping has also identified several important loci for high grain iron in rice, wheat and maize. In rice, QTL have been identified on chromosomes 1 and 5, each with high additive effects (Anuradha et�al. 2012). Candidate genes encoded on these loci include NA synthase *OsNAS3* and the iron transporter *OsYSL1*. In wheat, QTL on chromosomes 7DS and 4A have been identified that explain 14.5% and 21% of grain iron concentration variance, respectively (Crespo-Herrera et�al. 2016, Crespo-Herrera et�al. 2017). In maize one QTL on chromosome 5 that accounted for over 16% of variation in grain iron concentration has been mapped (Jin et�al. 2013).

QTL associated with both iron and zinc are not surprising, since chelators involved in mineral translocation, such as NA, bind both cations (Beneš et�al. 1983). Furthermore, iron transporters such as IRT1, considered the entry point for iron in many plant species, also transport zinc and other metals (Vert et�al. 2002). Since both high iron and high zinc are desirable traits for crops the combined QTL are helpful in breeding programs.

## Increasing Iron Using Transgenics

The discovery of genes specifically involved in plant iron homeostasis has naturally been seized upon to investigate if overexpression could raise iron levels, without yield penalties, either in specific tissues or in the whole plant. Genes in all steps of iron homeostasis—uptake, transport, storage and regulation—have been investigated to date, either individually or in combination (reviewed in Bashir et�al. 2013b, Masuda et al. 2013a, Vasconcelos et�al. 2017, Kawakami and Bhullar 2018). The large number of studies to date, including studies using the same genes but with different promoters and in different crop varieties (Table�2), allows us to draw some generalizations and conclusions about the most effective transgenic strategy for iron biofortification.

**Table 2:** Genetic approaches leading to increased iron in staple crops

Promoter^a^	Gene^a^	Crop/variety	Fold change in transcript or metabolite^b^	Fold increase in iron (tissue)	References
Transporters and reductases for Fe uptake					
* ZmUBQ*	*OsIRT1*	Rice (*Japonica* cv. Dongjin)	10 � (roots and shoots +Fe)	1.1 (brown seed)	Lee and An (2009)
* OsACT1*	*OsYSL15*	Rice (*Japonica* cv. Dongjin)	>100 � (leaves)	1.3 (brown seed)	Lee et al. (2009a)
Biosynthesis of organic Fe chelators and their transport					
* CaMV 35S*	*OsNAS1*	Rice (*Japonica* cv. Nipponbare)	6 � NA (seed)	2 (brown seed)	Johnson et al. (2011)
				2 (polished)	
* CaMV 35S*	*OsNAS2*	Rice (*Japonica* cv. Nipponbare)	9 � NA (seed)	3 (brown seed)	Johnson et al. (2011)
				4 (polished)	
* CaMV 35S*	*OsNAS3*	Rice (*Japonica* cv. Nipponbare)	11 � NA (seed)	2 (brown seed)	Johnson et al. (2011)
				2 (polished)	
* CaMV 35S*	*OsNAS3*	Rice (*Japonica* cv. Dongjin)	10 � NA (seed)	3 (brown seed)	Lee et al. (2009b)
				2 (polished)	
* ZmUBQ*	*OsNAS2*	Wheat cv. Bobwhite	3 � NA (grain)	1.3 (grain)	Beasley et al. (2019)
			2 � NA (white flour)	1.5 (white flour)	
* OsACTIN1*	*HvNAS1*	Rice (*Japonica* cv. Tsukinohikari)	15 � NA (shoots)	2.5 (polished)	Masuda et al. (2009)
* CaMV 35S*	*HvNAS1*	Soybean	3 � NA (seeds)	4 (seeds)	Nozoye et al. (2014)
* CaMV 35S*	*HvNAS1*	Sweet potato	7 � NA (leaves)	2 (storage roots)	Nozoye et al. (2017)
* HvIDS3*	*HvIDS3*	Rice (*Japonica* cv. Tsukinohikari)		1.3 (brown seed)	Masuda et al. (2008)
				1.4 (polished)	
* OsSUT1*	*OsYSL2*	Rice (*Japonica* cv. Tsukinohikari)	6 � (ear)	4 (polished seed)	Ishimaru et al. (2010)
Regulators					
* CaMV 35S*	*OsIRO2*	Rice (*Japonica* cv. Tsukinohikari)	>60 � (seedlings)	3 (brown seed)	Ogo et al. (2011)
T-DNA insertion	*OsHRZ1*	Rice (*Japonica* cv. DongJing)	0.7 � (roots)	1.7 (brown seed)	Kobayashi et al. (2013)
RNAi line	*OsHRZ2*	Rice (*Japonica* cv. Tsukinohikari)	0.6 � (roots)	3.5 (brown seed); 3 (polished seed)	Kobayashi et al. (2013)
Fe storage					
* OsGLUB1*	*GmFERH1*	Rice (*Japonica* cv. Kitaake; Indica cv. IR68144)		3 (brown seed)	Goto et al. (1999) and Oliva et al. (2014)
				2 (polished)	
* OsGLB1, OsGLUB1*	*GmFERH1*	Rice (*Japonica* cv. Kitaake)		1.3 (brown)	Qu et al. (2005)
* OsGLUA2*	*OsFER2*	Rice (Indica cv. Pusa-Sugandh II)	4.1–7.8 � (polished seed)	2.1 (polished)	Paul et al. (2012)
* Zm27gZEIN*	*GmFERH1*	Maize (B73)	10 � *GmFERH1* vs. native *FER*	1.2 (seed)	Kanobe et al. (2013)
* OsGLUB*	*PvFER1*	Wheat cv. Bobwhite		1.6 (grain)	Singh et al. (2017b)
* TaGLU-1D-1*	*TaFER1-A*	Wheat cv. Bobwhite	50–200 � (endosperm)	1–1.5 (grain)	Borg et al. (2012) and Neal et al. (2013)
* TaGLU-1D-1*	*TaVIT2-D*	Wheat cv. Fielder	10 � (grain)	2 (white flour)	Connorton et al. (2017b)
	*OsVIT1 or OsVIT2*	Rice (*Japonica* cv. Dongjin)	<0.2 � *OsVIT1* or *OsVIT2*	1.4 (brown seed)	Zhang et al. (2012)
				1.8 (polished)	
* StPATATIN1*	*AtVIT1*	Cassava		3–4 (tuber)	Narayanan et al. (2015)
Transgene combinations					
* ZmUBQ1:OsNAS1 + ZmUBQ1:HvNAATb*		Rice (EYI 105)	160 � NA, 29x DMA (seeds)	4 (polished)	Banakar et al. (2017a)
* OsSUT1:OsYSL2 + OsGLUB1:GmFERH2 + OsGLB:OsYSL2 + OsACT1:HvNAS1 + OsGLB:GmFerH2*		Rice (*Japonica* cv. Tsukinohikari; *Japonica* cv. Paw San Yin)	3 � NA, 6 � DMA (brown seeds)	1.5 (brown Tsukinohikari), 3 (polished Tsukinohikari), 3.4 (polished Paw San Yin)	Masuda et al. (2012) and Aung et al. (2013)
* OsGLB:GmFERH2 + HvNAAT-A,-B + HvIDS3*		Rice (*Japonica* cv. Tsukinohikari)		2 (polished)	Masuda et al. (2013b)
* CaMV35S:OsNAS2 + OsGLUA2:GmFERH1*		Rice (Indica IR64)	10 � *OsNAS2* (leaves)	6 (polished)	Trijatmiko et al. (2016)
* MsENOD12B:AtIRT1 + OsGLUB:PvFER1 + 35S:AtNAS1*		Rice (*Japonica* cv. Taipei 309)		4 (polished)	Wirth et al. (2009) and Boonyaves et al. (2016)
* AtA14:AtIRT1 + StPATATIN1:AtFER1*		Cassava		5.5 (tuber)	Narayanan et al. (2019)

^a^Plant species are abbreviated as follows: *At, Arabidopsis thaliana*; *Gm*, *Glycine max* (soybean); Hv, *Hordeum vulgare* (barley); *Ms*, *Medicago sativa*; *Os*, *Oryza sativa* (rice); *St*, *Solanum tuberosum* (potato); *Ta*, *T. aestivum* (wheat).

^b^Fold change compared with endogenous transcript. For transgenes from a different species, no value is given. For plants overexpressing the *NAS* genes, the fold change in NA is given.

The iron in the plant-based foods we consume has been handled by many plant proteins before it ends up as a metal cofactor or in a storage form (see Kawakami and Bhullar 2018 for a summary in rice). First, enzymes such as proton ATPases, ferric reductases and those for the synthesis of secreted coumarins and phytosiderophores mediate the solubilization of iron hydroxides in the soil. Next, iron is transported from the apoplast to the symplast, mediated primarily by IRT1 in dicots and proteins of the Yellow Stripe (YS) family in grasses (Fig.�1). Numerous transporters are required for the distribution of iron within the plant through the xylem and phloem. Inside cells, specific transporter proteins facilitate transport across intracellular membranes, and biosynthetic enzymes incorporate iron into iron–sulfur clusters or heme. Alternatively, iron is stored in vacuoles or in ferritin in the plastids. Any of these proteins/genes could be of interest as biofortification targets.

### Increasing iron uptake

The uptake of iron involves a plethora of genes (Brumbarova et�al. 2015, Connorton et�al., 2017a) and only a handful of them have thus far been explored as biofortification targets. Plants have two main strategies for iron uptake, the chelate-based strategy in grasses and reductive strategy in non-grasses. Overexpression of *IRT1*, a divalent metal transporter central to reductive iron uptake, increased the iron concentration in rice leaves by 1.7-fold but only by 1.1-fold in grains (Lee and An 2009). These findings suggest that in the absence of extra sink capacity in the seeds, iron accumulates in the vegetative tissues. Indeed, when *IRT1* is overexpressed together with *PvFER1* in the endosperm, the iron concentration increased up to 4-fold in polished rice (Boonyaves et�al. 2017). In cassava, the combined overexpression of Arabidopsis *IRT1* and *FER1* resulted in 5.5-fold more iron in tubers (Narayanan et�al. 2019). The reductive iron uptake mechanism also involves secretion of small molecules, such as coumarin-derivatives in some species and flavins in others (Connorton et�al. 2017a), but whether the biosynthesis genes can be used for biofortification has not been investigated to date. In grasses and cereals, derivatives of NA—mugineic acid and deoxymugineic acid (DMA)—are secreted in the rhizosphere where they chelate Fe^3+^. The Fe-chelator complexes are transported into the cell by YS1 in maize and YSL15 in rice (Curie et�al. 2001, Curie et�al. 2009). However, increasing DMA by overexpressing *IDS3* (Iron Deficiency Specific Clone 3, encoding 2'-deoxymugineic-acid 2'-dioxygenase) or overexpressing *YSL15* gave only modest increases in the iron concentration in rice seeds (Masuda et�al. 2008, Lee et�al. 2009a).

### Facilitating iron distribution

Iron is transported around the plant in a chelated form, mainly citrate and malate in the xylem, and NA and its derivatives in the phloem. The Fe-NA complexes are transported across membranes by YSL transporters, such as YSL2 in rice (Ishimaru et�al. 2010). Biofortification attempts have focused on NA, as it is specifically involved in the transport of divalent metals and not, like citrate, a more general metabolite. Also, the biosynthesis of NA requires only one enzymatic step, mediated by nicotianamine synthase (NAS), which uses S-adenosyl methionine as a substrate. The *NAS* genes have been overexpressed using strong constitutive promoters such as the Cauliflower Mosaic Virus *35S* and *ZmUBIQUITIN* promoters. This can increase NA levels more than 10-fold in leaves, or even higher in seeds, pushing up iron concentrations approximately 2-fold in grains of rice and wheat (Table�2). In grasses, NA is converted to DMA by nicotianamine aminotransferase (NAAT) and DMA synthase (DMAS; Bashir et�al. 2006). DMA is secreted by the roots to facilitate Fe^3+^ uptake (see above), but it also occurs in leaves. The combined overexpression of *NAS1* and *NAAT* in rice led to a 29-fold increase in DMA and 4-fold increase in iron concentration in polished rice (Banakar et�al. 2017b). Another approach to increase iron transport from the phloem into the developing seeds has been to overexpress the *YSL2* transporter in rice. Ishimaru et�al. (2010) reported that it was important to use the *OsSUT1* promoter to increase iron levels in polished rice, as no effect was seen with the *35S* promoter. The *SUT1* sucrose transporter is strongly expressed in phloem companion cells and immature seeds, and evidently the tissue-specific expression was important to load more iron into the grain endosperm. Increased levels of NA also led to higher zinc and manganese concentrations in the grain, because NA facilitates the mobilization of these divalent metals, among others (Curie et�al. 2009, Clemens et�al. 2013). Interestingly, NA also improves the bioavailability of iron, as discussed below.

### Enhancing iron storage

In plants, iron is stored in the form of ferritin or in vacuoles. Plant ferritin genes have been overexpressed in rice, wheat and maize, using endosperm-specific promoters (Table�2). Expression of the soybean *FERH1* gene in rice led to a 3-fold increase in iron concentration in unpolished or polished grains, but the same gene was less effective in maize. In wheat, only a 1.5-fold increase in iron concentration was found by expressing *FER1* from common bean (*P. vulgaris*) or from wheat itself (Borg et�al. 2012, Neal et�al. 2013, Singh et�al. 2017a). Assuming that all constructs are similarly effective in raising ferritin expression levels, it may be that rice is able to direct more iron to ferritin than wheat. Interestingly, overexpression of a vacuolar iron transporter, *TaVIT2*, with the same endosperm-specific *GLU-1D-1* promoter used for ferritin overexpression, raised iron levels more than 2-fold (Connorton et�al. 2017b). Enhancing vacuolar iron storage may be more effective than ferritin iron storage because the former is how iron is stored normally in cereal grains. Overexpression of *VIT* genes in rice has not been fully explored, but knockdown or knockout of *VIT1* or *VIT2* led to iron accumulation in the embryo, and increased the total iron concentration in brown rice by approximately 25–30% or 1.3-fold (Zhang et�al. 2012, Bashir et�al. 2013a). The iron concentration of roots and shoots of the *vit* mutant lines was lower than in wild type. Thus, decreased storage capacity for iron in these parts of the plant seemed to redirect iron to the seed and embryo (but not the endosperm). Overexpression of Arabidopsis *VIT1* in the tubers of cassava was also effective, raising iron 3- to 4-fold. Taken together, increasing the storage capacity of iron works well as a single-gene strategy, but whether ferritin or vacuolar storage should be used may differ from species to species.

In addition to targeting one aspect of iron homeostasis, multigene approaches to simultaneously increase iron uptake, distribution and storage have been very successful (Table�2). Another way of changing the expression of several genes would be to interfere with global regulators of iron homeostasis. For example, overexpression of the transcription factor *OsIRO2*, which activates a number of genes for iron uptake, resulted in a 3-fold increase in the iron concentration in brown rice (Ogo et�al. 2011). Other promising targets are the *HRZ1* and *HRZ2* genes, which act as negative regulators of the transcriptional response to iron deficiency. Rice mutants in *HRZ1* or *HRZ2* have constitutively induced transcript levels of genes for iron uptake and mobilization (Kobayashi et�al. 2013), and they accumulate 1.7- to 3.5-fold iron in seeds. Although seed viability is diminished in *hrz1* mutant lines, perhaps when combined with increased iron storage capacity this could be an efficient biofortification strategy in the future.

## Improving Iron Bioavailability

Bioavailability broadly refers to the proportion of a nutrient that is absorbed from the diet and used for normal body functions. As already mentioned, the bioavailability of iron is low (<15%) in plant foods, and therefore an important factor to consider in biofortification approaches of staple crops. Bioavailability is not straightforward to measure, and depends not only on external factors such as the food matrix and the chemical form of the nutrient, but also on internal factors including gender, age, nutrient status and life stage (e.g. pregnancy) (Hurrell and Egli 2010). Showing that biofortification of a certain crop leads to better iron nutrition requires human intervention studies which can be demanding logistically and come at considerable costs. As a proxy for bioavailability, the absorption of iron into Caco-2 cells can be measured (Glahn et�al. 1998). This is a human cell line that morphologically and functionally resembles the enterocytes lining the small intestine, except that they are not protected by a mucosal layer. Iron absorption is measured by the amount of human ferritin that is formed in response to added iron sulfate (positive control) or a digested food matrix (Glahn et�al. 1998). The in vitro Caco-2 cell assays can also reveal the inhibitory effects of antinutrients such as phytic acid. Animal studies of iron bioavailability have been performed using mice or chickens, but these models do not fully recapitulate nutrient absorption in the human gut. The biophysical measure of iron bioaccessibility, which is the potential availability of a mineral in food for absorption in the gut following digestion, may also provide some indication of bioavailability (Narayanan et�al. 2019).

While the chemical form (speciation) of iron affects its bioavailability, the speciation of iron in plant foods is dramatically altered by food preparation (cooking) and intestinal digestion. For example, Fe(III)hydroxides in ferritin are released as Fe^2+^ or Fe^3+^ during heating (Hoppler et�al. 2008), and likely chelated by phytic acid in the food matrix (Moore et�al. 2018, Perfecto et�al. 2018). Overall, the amount of phytic acid is a strong indicator for bioavailability, and in the absence of any enhancers of iron absorption a molar ratio of phytic acid: iron of <1:1 is required for significantly improved absorption of iron from cereal-based meals (Hurrell and Egli 2010). In some pulses and wholegrain cereals, the molar ratio is ≥10:1 and bioavailability is effectively zero in Caco-2 cell culture (Rodriguez-Ramiro et�al. 2017), but a fractional absorption of 4–6% iron was measured in young women by isotope studies (Petry et�al. 2013). Efforts to breed crops with lower phytic acid have been reported, although a strong decrease in phytic acid may affect yield. A 60% decrease in phytic acid in pea was shown to improve iron bioavailability in Caco-2 cell studies (Warkentin et�al. 2012, Liu et�al. 2015) and common bean carrying the *low phytic acid* (*lpa*) trait improved the iron status of young females (Petry et�al. 2013). Alternatively, phytic acid has been lowered by expressing a fungal phytase resulting in 3-fold more bioavailable iron in maize (Drakakaki et�al. 2005). In cereal grains, phytic acid is mostly localized to the bran, in contrast to low levels in the endosperm (Fig.�1). Therefore, targeting iron specifically to the endosperm is an alternative solution to the ‘phytate problem’ (Connorton et�al. 2017b).

Differences in iron bioavailability are not exclusively attributable to differences in phytic acid levels. QTL controlling total grain iron barely overlapped with bioavailability QTL and combining three of the larger QTL led to higher iron bioavailability (Lung’aho et�al. 2011). In parallel, several independent studies have shown that increasing the NA content, either by overexpressing *NAS* genes in rice and wheat (Zheng et�al. 2010, Beasley et�al. 2019) or by adding NA to the food matrix (Zheng et�al. 2010), enhances bioavailability of iron in mice or in Caco-2 cell culture. However, human intervention studies have thus far not been conducted.

## Concluding Remarks

Both conventional breeding and transgenic approaches have shown that it is possible to increase iron concentrations in staple crops. More than 10 years of transgenic approaches have revealed useful lessons for future developments of biofortification. For example, it has taught us that increasing the uptake of iron from the soil needs to be combined with increased iron storage capacity. Whether vacuolar or ferritin iron storage should be targeted depends on the plant species and tissue. Promoters and genes, or gene paralogs, appear to be equally effective in different crop species. Also, it has been observed that some transgenic strategies increase iron specifically, whereas others (e.g. *NAS* genes) increase both iron and zinc levels. And finally, an important finding is that increased biosynthesis of NA also improves the bioavailability of iron.

Doubtlessly more gene targets remain to be explored. In particular, rather than overexpressing several transgenes, manipulating a single regulatory gene either by changing expression levels or by modulating its activity, may achieve a similar transcriptional response. Studies on *IRO2* and the *HRZ* genes in rice are the start of this approach, and further gene targets may be identified by forward genetics approaches such as QTL mapping or GWAS. While forward genetic studies in the model crop Arabidopsis could also be useful for gene discovery, this should then focus on iron homeostasis in seeds and not other tissues. In addition, the proteins that mediate iron transfer from the maternal tissues to the embryo are little known, and their overexpression could greatly enhance iron loading of seeds.

It is important for plant scientists to work together with nutritionists, to test iron bioavailability of biofortified crops in in vitro studies or in human intervention studies. Discussions with researchers involved in implementation of high-iron crops may help direct the research toward particular crops or specific plant foods. And ultimately, a broader discussion with the public about ‘nutritious foods’ is required to increase the acceptance of iron-biofortified crop varieties in addition to existing practices of chemical fortification and iron supplements. In particular for rice and wheat, transgene expression or gene editing are necessary to achieve sufficient iron levels in a meat-free diet. Encouragingly, genetically modified crops are already accepted and grown in several countries.
